# The RecA-NT homology motif in ImuB mediates the interaction with ImuA′, which is essential for DNA damage–induced mutagenesis

**DOI:** 10.1016/j.jbc.2024.108108

**Published:** 2024-12-18

**Authors:** Joana A. Santos, Kęstutis Timinskas, Atondaho A. Ramudzuli, Meindert H. Lamers, Česlovas Venclovas, Digby F. Warner, Sophia J. Gessner

**Affiliations:** 1 Department of Cell and Chemical Biology, Leiden University Medical Center, Leiden, The Netherlands; 2 Institute of Biotechnology, Life Sciences Center, Vilnius University, Vilnius, Lithuania; 3 SAMRC/NHLS/UCT Molecular Mycobacteriology Research Unit, DSI/NRF Centre of Excellence for Biomedical TB Research, Department of Pathology, University of Cape Town, Cape Town, South Africa; 4 Institute of Infectious Disease and Molecular Medicine, University of Cape Town, Cape Town, South Africa; 5 Wellcome Centre for Infectious Diseases Research in Africa, University of Cape Town, Cape Town, South Africa

**Keywords:** *Mycobacterium tuberculosis*, protein–protein interaction, mutagenesis, DNA repair, antibiotic resistance, mutasome

## Abstract

The mycobacterial mutasome—comprising ImuAʹ, ImuB, and DnaE2—has been implicated in DNA damage–induced mutagenesis in *Mycobacterium tuberculosis*. ImuB, which is predicted to enable mutasome function *via* its interaction with the β clamp, is a catalytically inactive Y-family DNA polymerase. Like some other members of the Y-family, ImuB features a recently identified amino acid motif with homology to the RecA N terminus (RecA-NT). Given the role of RecA-NT in RecA oligomerization, we hypothesized that ImuB RecA-NT mediates the interaction with ImuAʹ, an RecA homolog of unknown function. Here, we constructed a panel of *imuB* alleles in which the RecA-NT was removed or mutated. Our results indicate that RecA-NT is critical for the interaction of ImuB with ImuAʹ. A region downstream of RecA-NT, ImuB-C, appears to stabilize the ImuB–ImuAʹ interaction, but its removal does not prevent complex formation. In contrast, replacing two hydrophobic residues of RecA-NT, L378 and V383, disrupts the ImuAʹ–ImuB interaction. To our knowledge, this is the first experimental evidence suggesting a role for RecA-NT in mediating the interaction between a Y-family member and an RecA homolog.

The mycobacterial mutasome, which has been implicated in DNA damage tolerance and induced mutagenesis in the major human pathogen, *Mycobacterium tuberculosis*, is composed of an RecA homolog (ImuAʹ), a catalytically inactive Y-family DNA polymerase (ImuB), and an error-prone C-family DNA polymerase (DnaE2) (1, 2, 3, 4). Although experimental evidence supports the role of DnaE2 as an error-prone translesion synthesis (TLS) polymerase (1), the functions of the ImuAʹ and ImuB accessory proteins remain elusive. ImuB lacks the acidic amino acids required for DNA polymerase activity but contains a functional β clamp-binding motif that is thought to mediate access of DnaE2 (which lacks a β clamp-binding motif) to DNA. In support of this hypothesis, mutations in the ImuB β clamp-binding motif abolish DNA damage–induced mutagenesis (2) and abrogate ImuB-β colocalization *in vivo* (5); however, the precise molecular function of ImuB is not known. The function of ImuAʹ is also unclear, but homology to RecA makes it tempting to postulate a structural role similar to that of RecA in binding components of a mutagenic complex and regulating its activity (2, 6, 7). Notably, recent work has demonstrated that *Myxococcus xanthus* ImuA possesses ATPase activity thought to play a role in mutasome assembly and disassembly (8). The myxococcal ImuA protein has also been implicated in inhibition of recombination repair by directly binding RecA, thereby facilitating a switch from error-free repair to TLS (9).

The central role of ImuB can be ascribed to its multiprotein binding capacity, which is facilitated by its C-terminal region. A yeast two-hybrid assay suggested that ImuB can interact with ImuAʹ, DnaE2, and itself, as well as the *dnaE1*-encoded high-fidelity replicative DNA polymerase (2). An attempt in the same study to identify an ImuB interaction region at the N terminus (first 48 residues) of ImuAʹ failed, whereas a C-terminal deletion (40 residues) seemed to abolish interaction with ImuB. However, one major limitation of the yeast two-hybrid system is the inability to rule out the loss of structural integrity consequent on the engineered mutant alleles as a cause of failed interactions (2). A recent *in vitro* study using myxococcal ImuA and ImuB suggested that these two proteins interact *via* a region that overlaps with the β clamp-binding motif using adenylate cyclase two-hybrid screen and microscale thermophoresis (8). However, that work was limited to *in vitro* analyses, used a truncated version of ImuB, and identified only a general region implicated in the interaction (residues 339–369).

Recently, a motif was identified in the C-terminal regions of some active and inactive Y-family polymerases that resembles the N-terminal region of *Escherichia coli* RecA (RecA-NT) (7). This RecA-NT motif enables binding to the neighboring RecA molecule, facilitating oligomerization and the formation of a nucleoprotein filament on single-stranded DNA. Computational structural modeling suggested that the RecA-NT motif in *E. coli* UmuC—an essential subunit of the paradigmatic Y-family TLS DNA polymerase, pol V—may mediate the interaction between UmuC and RecA in an analogous fashion (7). Strong support for the proposed binding mode was provided by experimental data (10). Furthermore, a large-scale sequence analysis revealed that this type of interaction between RecA and Y-family polymerases might be ubiquitous, including catalytically inactive Y-family members such as ImuB given the presence of the RecA-NT motif at the C terminus of the Y-family polymerases (7). Based on the homology between ImuAʹ and RecA, it might be expected that the RecA-NT motif in ImuB mediates binding to ImuAʹ (7).

Here, we used computational modeling to derive a putative structure of the complex between mycobacterial ImuAʹ and ImuB proteins and to evaluate potential features underlying their interaction. Based on the model, we identified regions and specific residues in the RecA-NT motif predicted to be key in maintaining a stable interaction between ImuAʹ and ImuB. Using this information, we designed mutations to disrupt the interaction interface, then assessed their impact *in vitro* in biochemical assays and *in vivo* in phenotypic complementation experiments utilizing a Δ*imuA*ʹΔ*imuB* double deletion mutant of *Mycobacterium smegmatis*, a mycobacterial model organism (2). Our results indicate that RecA-NT is essential to secure a stable ImuAʹ–ImuB interaction and, consequently, mutasome function. Truncation of the RecA-NT motif or substitution of a pair of key hydrophobic residues at the predicted ImuAʹ–ImuB interface abolishes mutasome function.

## Results

### Computational modeling of the ImuAʹ–ImuB interaction

**Figure 1:**
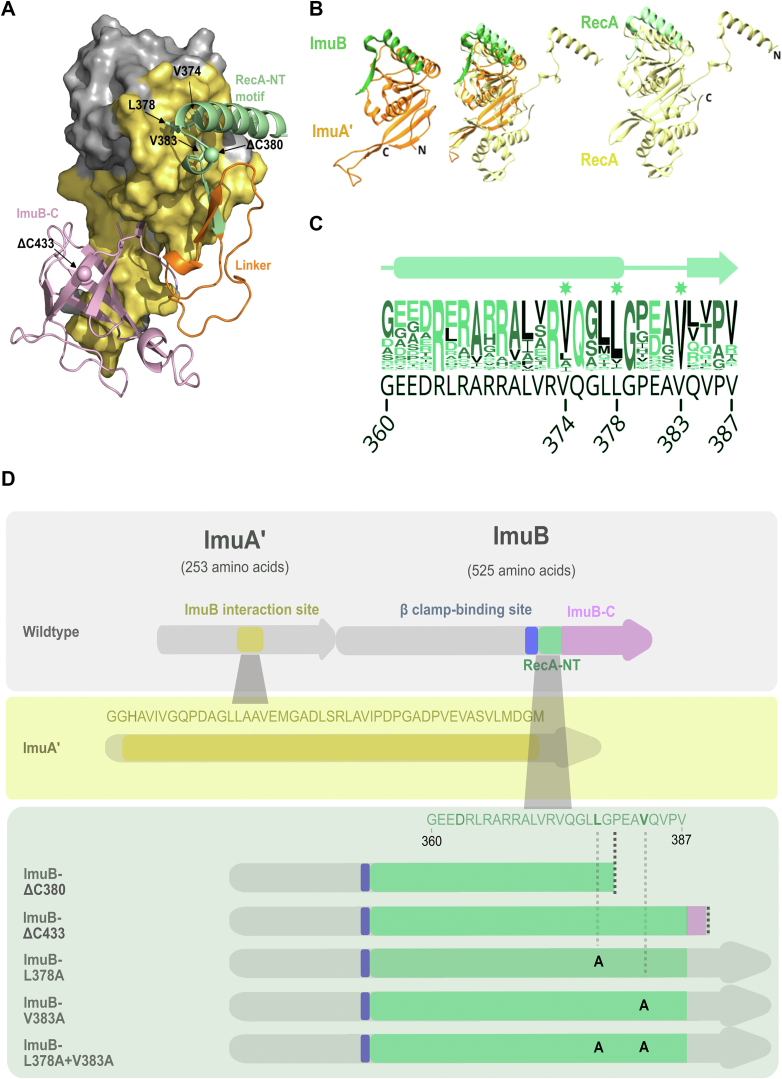
**Computational model of mutasome interactions.***A*, AlphaFold model of the ImuAʹ–ImuB complex. The ImuAʹ structure is shown as Connolly (solvent excluded) surface. For ImuB, only the C-terminal region is shown in the *cartoon representation*. The ImuAˈ surface in contact with ImuB is colored in *yellow*. *Green* represents the RecA-NT motif in ImuB (residues 360–387), *pink* represents the ImuB-C (residues 422–525), and *orange* indicates the linker between the two (residues 388–421). *Spheres* mark the residues at which C-terminal truncations of ImuB were made. ImuB residues predicted to be necessary for the ImuAʹ–ImuB interaction are labeled and represented in *sticks* (V374, L378, and V383). *B*, comparison of ImuAʹ–ImuB model (*left*) with X-ray structure of the RecA–RecA complex (*right*). Superposition of the two structures is shown in the *middle*. For ImuB and the second RecA, only the RecA-NT motif (*shades of green*) is shown. RecA dimer was obtained from the structure of RecA filament (Protein Data Bank ID: 3CMW, (31)). *C*, conservation logo of RecA-NT derived from the aligned mycobacterial ImuB homologs with the corresponding *Mycobacterium smegmatis* ImuB sequence added below (in *black*). Numbering corresponds to *M. smegmatis* sequence. Individual residues selected for site-directed mutagenesis are indicated with a star above the logo. *D*, *M. smegmatis imuAʹimuB* operon with predicted interaction sites. In ImuAʹ, the predicted ImuB interaction site stretches from G97 to M144. In ImuB, the β clamp-binding motif (352-QLPLW-356) is followed by RecA-NT (G360-V387) and ImuB-C (P422-E525). The *panels* below indicate the site-directed mutations and ImuB C-terminal deletion mutants constructed in this study.

We used AlphaFold (11, 12) to generate a structural model of the ImuAʹ–ImuB complex. Figure 1*A* shows part of the model consisting of the C-terminal region of ImuB (ImuB residues 360–525) including the RecA-NT homology motif bound to ImuAʹ. Comparison of our AlphaFold model of ImuA′–ImuB–RecA-NT with the crystal structure of RecA–ssDNA filament (Fig. 1*B*) reveals that the two protein complexes are organized in a similar manner, despite their low sequence conservation. Sequence analysis of mycobacterial ImuB sequences reveals several conserved residues in the RecA-NT domain (Fig. 1*C*) that are located at the predicted interaction surface with ImuA' (Fig. 1*A*). Using this information, we predicted that residues V374, L378, and V383 might play an important role in ImuAˈ–ImuB complex formation based on their position at the interaction interface as well as positional and sequence conservation (Fig. 1, *C* and *D*).

### The ImuB RecA-NT motif is essential for interaction with ImuA′

**Figure 2:**
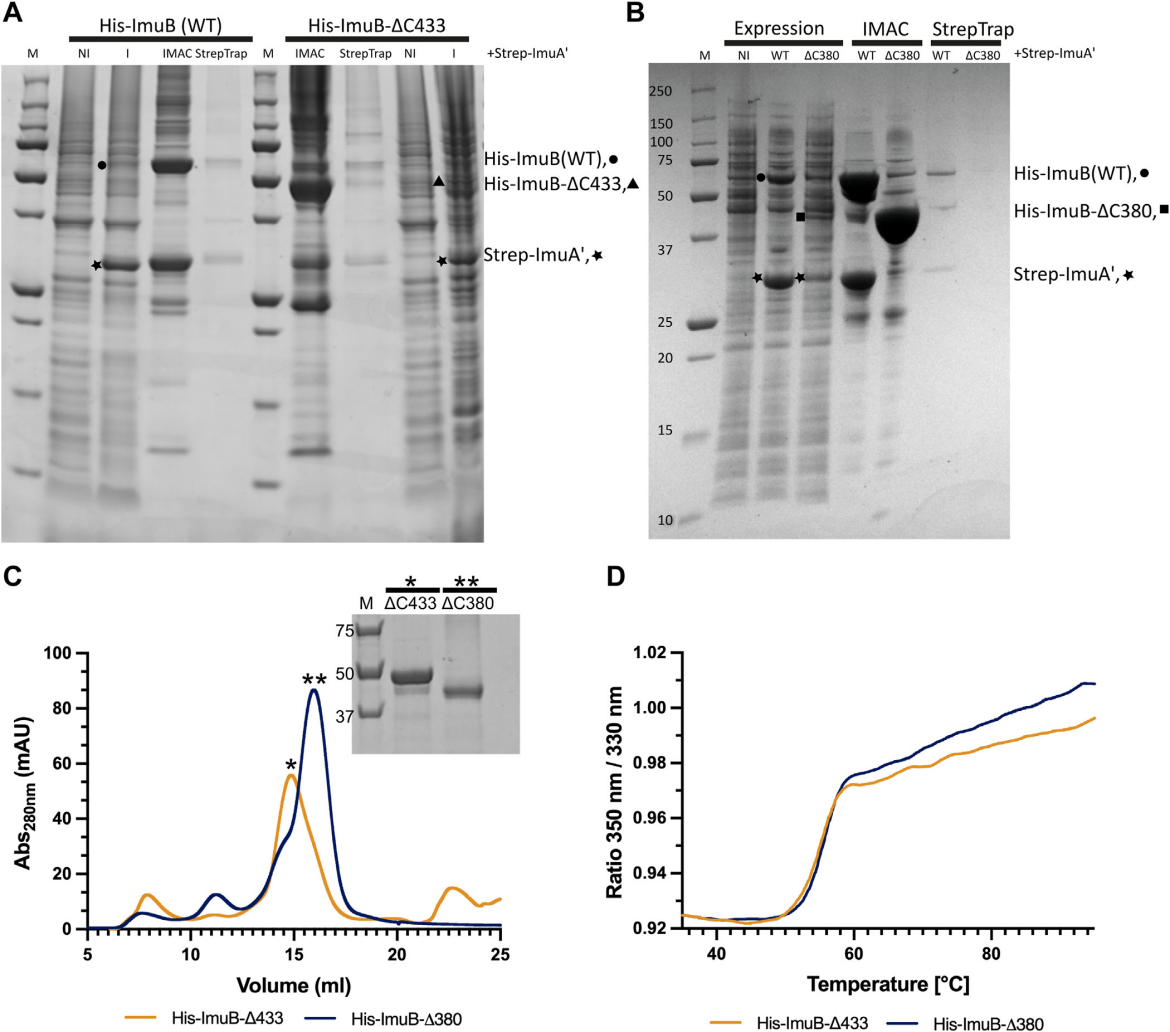
**The C terminus of ImuB is essential for ImuAʹ–ImuB complex formation *in vitro*.***A*, SDS-PAGE analysis of elution samples from two consecutive pull-downs. Following coexpression of Strep-ImuAʹ (★) with either His-ImuB (WT, •) or His-ImuB-ΔC433 (▲), the cell-lysate supernatant was loaded onto a HisTrap column (IMAC) and eluted with an imidazole gradient. Eluted fractions were subsequently loaded on a StrepTrap column, and Strep-ImuAʹ was eluted with desthiobiotin. *B*, SDS-PAGE analysis of coexpression of Strep-ImuA′-His-ImuB, Strep-ImuAʹ, and His-ImuB-ΔC380. Expression samples correspond to the total expression after growth for 3 h following IPTG induction. Bands corresponding to individual induced proteins are marked His-ImuB (•), Strep-ImuAʹ (★), and His-ImuBΔC380 (∎). *C*, SEC profiles of His-ImuB-ΔC433 and His-ImuB-ΔC380 following an IMAC. The *inset* shows the SDS-PAGE analysis of the peak fractions of both His-ImuB-ΔC433 (∗) and His-ImuB-Δ3C80 (∗∗). *D*, melting curves of His-ImuB-ΔC433 and His-ImuB-ΔC380 following SEC. His-ImuB-ΔC433 has a melting temperature of 55.1 °C and His-ImuB-ΔC380 of 55.6 °C. IMAC, immobilized metal affinity chromatography; SEC, size-exclusion chromatography.

To validate the AlphaFold model of the ImuA′–ImuB complex, we performed biochemical assays utilizing purified recombinants of wildtype ImuAʹ and ImuB and two C-terminal deletion constructs of ImuB. Previously, we showed that coexpression of ImuAʹ and ImuB produces a stable complex, so that His-tagged ImuB can be used to pull down Strep-tagged ImuAʹ (*via* immobilized metal affinity chromatography [IMAC]; HisTrap) and, reciprocally, Strep-tagged ImuAʹ can pull down His-ImuB (*via* affinity chromatography, StrepTrap) (5). To assess the contribution of ImuB-C-terminal to the interaction with ImuAʹ, two truncated versions of His-tagged ImuB were studied: His-ImuB-ΔC380 (retaining only part of RecA-NT) and His-ImuB-ΔC433 (which retains RecA-NT but lacks the ImuB-C region). The two truncations were individually coexpressed with Strep-tagged ImuAʹ (Strep-ImuAʹ) in *E*. *coli*, and interactions were tested over two consecutive pull-downs (Fig. 2). Similar to wildtype His-ImuB, His-ImuB-ΔC433 coeluted with Strep-ImuAʹ over the two pull-downs, indicative of complex formation between the two proteins (Fig. 2*A*). However, His-ImuB-ΔC380 was unable to pull-down Strep-ImuAʹ and, therefore, was absent in the subsequent pull-down using Strep-tagged ImuAʹ (Fig. 2*B*). In combination, these results support the inferred requirement of the intact ImuB RecA-NT motif for functional interaction with ImuAʹ.

To confirm that loss of interaction was not caused by altered folding or stability owing to deletion of the C-terminal region, we analyzed both ImuB-ΔC433 and ImuB-ΔC380 by size-exclusion chromatography (SEC) (Fig. 2*C*) and thermal protein melt (13) (Fig. 2*D*). SEC, which evaluates the oligomeric state and aggregation, showed that both ImuB-ΔC433 and ImuB-ΔC380 were eluted as single major peaks with elution volumes of 16 ml and 15 ml, respectively, which reflects their 10 kDa difference in molecular weight (Fig. 2*C*). SDS-PAGE analysis of the peak fraction confirmed that the eluted proteins had the expected molecular sizes of 50 kDa for ImuB-Δ433 (∗) and 40 kDa for ImuB-ΔC380 (∗∗) (Fig. 2*C*). Thermal melt assays measure the changes in melting (denaturation) temperatures and, by extension, protein stability. This was determined using differential scanning fluorimetry, relying on the intrinsic fluorescence of native tryptophan and tyrosine residues. A change in fluorescence profile over a temperature gradient signals a change in protein stability (13). Here, ImuB-ΔC433 and ImuB-ΔC380 showed almost superimposable melting curves that yielded similar melting temperatures: 55.1 °C for His-ImuB-ΔC433 and 55.6 °C for His-ImuB-ΔC380 (Fig. 2*D*), demonstrating equivalent thermostability of the two truncations. Together, these results suggest that the observed loss of interaction between His-ImuB-Δ380 and Strep-ImuAʹ was unlikely a consequence of impaired folding or stability.

**Figure 3:**
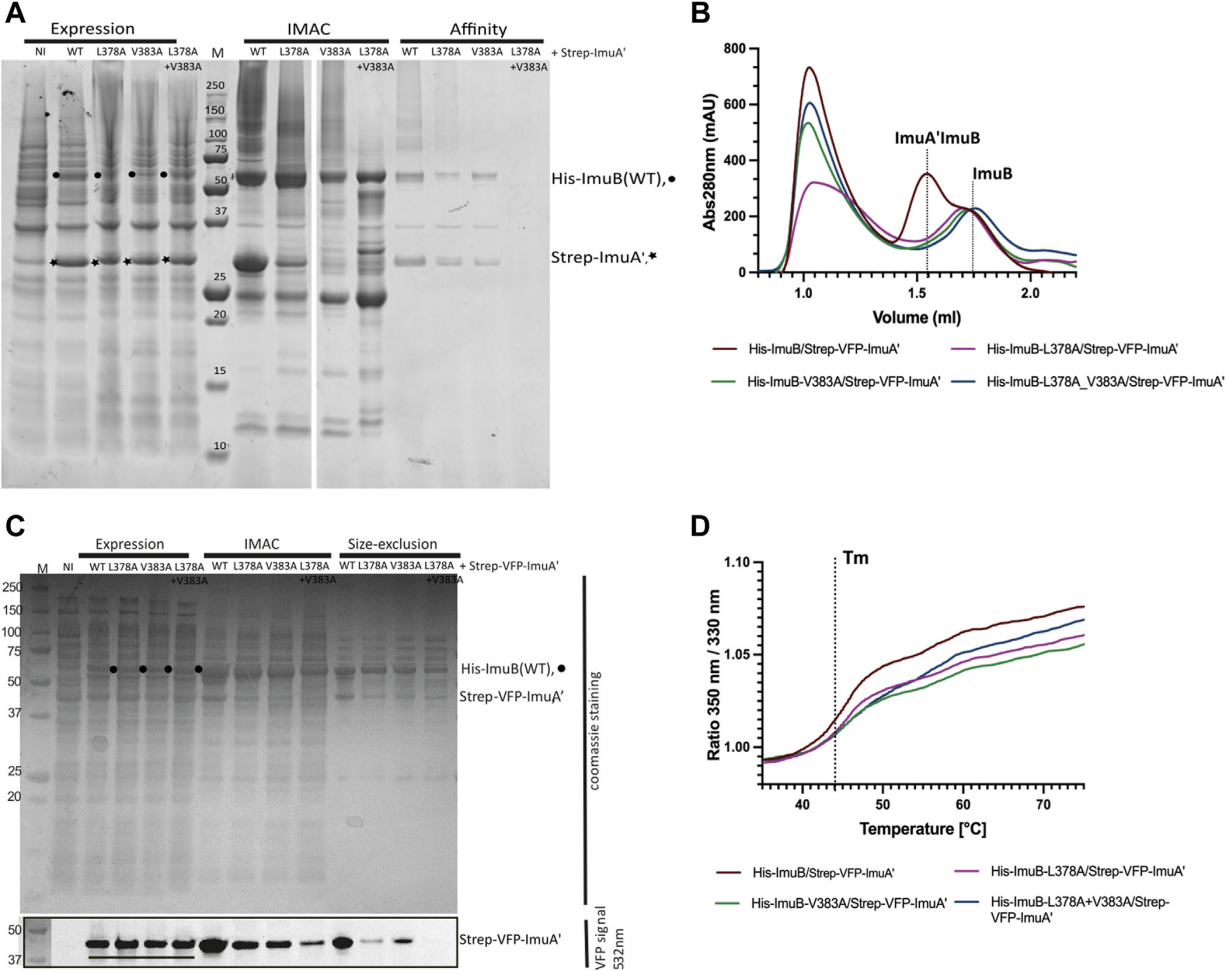
**Conserved hydrophobic residues of the Rec-NT motif are important for ImuA′–ImuB binding.***A*, SDS-PAGE analysis of the purification of His-ImuB mutants (ImuB-L378A, ImuB-V383A, and ImuB-L378A + V383A) with Strep-ImuAʹ after two sequential pull-downs with HisTag and StrepTag, respectively. Clarified extracts were loaded onto a HisTrap (IMAC) column and eluded with an imidazole gradient. Fractions containing His-ImuB were pooled, loaded into a StrepTrap column, and eluted with 5 mM desthiobiotin. Expression samples correspond to the total expression after growth for 3 h following induction with IPTG, and bands of individual induced proteins are marked His-ImuB (•) and Strep-ImuAʹ (★). *B*, SEC profiles of samples of His-ImuB and His-ImuB mutants (ImuB-L378A, ImuB-V383A, and ImuB-L378A + V383A) coexpressed with Strep-VFP–ImuAʹ following IMAC purification. The elution volumes for His-ImuBAʹ complexes and His-ImuB are highlighted with *dotted lines*. *C*, SDS-PAGE analysis of His-ImuB mutants coexpressed with VFP-Strep-ImuAʹ after IMAC and subsequent SEC. Clarified extracts were incubated with nickel beads and eluted with 500 mM imidazole. The fraction containing most of ImuB was injected into a S200 increase 2.4 ml column for SEC. Expression samples correspond to the total expression after growth for 3 h following induction with IPTG and are marked with (•) for His-ImuB and underlined (−−) for Strep-VFP–ImuAʹ. The *top panel* is the SDS-PAGE gel stained with Coomassie, and below is the same gel imaged at 532 nm for detection of the VFP fluorescent tag in ImuAʹ. The *bottom panel* corresponds to a crop of the gel and did not eliminate any bands. The marker lane added in the *right-hand side* of the *bottom panel* is the same as the one in the *top panel* and was properly aligned by superposition of the two images of SDS-PAGE gel. *D*, melting curves of His-ImuB and His-ImuB mutants (ImuB-L378A, ImuB-V383A, and ImuB-L378A + V383A) coexpressed with Strep-VFP–ImuAʹ following IMAC purification. The inflection point corresponding to the melting temperatures is marked with a *dotted line*. The melting temperatures measured were 45.1 °C for His-ImuB, 45.2 °C for His-ImuB-L378A, 45.4 °C for His-ImuB-V383A, and 45.9 °C for His-ImuB-L378A + V383A. IMAC, immobilized metal affinity chromatography; SEC, ssize-exclusion chromatography.

To further evaluate the roles of individual residues located at the interface of the ImuAʹ–ImuB AlphaFold model, mutant alleles were generated, and their effect on protein–protein interaction and mutasome function was investigated. Three ImuB amino acid residues within the RecA-NT homology motif were identified as part of the hydrophobic core of the interaction with ImuAʹ: V374, L378 (end of the α-helix), and V383 (start of the β-strand) (Fig. 1, *A* and *C*). Attempts to construct a V374A mutant failed, but V383A and L378A substitutions were successfully generated. To investigate the impact of the L378A, V383A, and combined L378A + V383A mutations on ImuAʹ–ImuB complex formation *in vitro*, we adopted the approach described earlier for the C-terminal truncations of ImuB. Whereas wildtype ImuB, ImuB-L378A, and ImuB-V383A eluted in complex with ImuAʹ, neither the ImuB-L378A + V383A nor ImuAʹ could be eluted *via* affinity chromatography (StrepTrap column) (Fig. 3*A*).

To ensure that impaired interaction was not a consequence of loss of folding or stability, we compared the ImuB mutants with wildtype ImuB by analyzing ImuAʹ–ImuB complexes using SEC and thermal protein melt (Fig. 3, *B* and *C*). ImuB was analyzed in complex with ImuAʹ rather than by itself because we previously demonstrated that, unlike the truncated versions, ImuB-ΔC433 and ImuB-ΔC380, full-length ImuB is susceptible to degradation in the absence of ImuA' (5). At the same time, we ensured the detection of ImuAʹ in fractions collected after SEC by using the Strep-VFP-tagged version of ImuAʹ as described (5), which allows visualization of ImuAʹ at 532 nm in the SDS-PAGE analysis (Fig. 3*B*). Notably, our prior work established that addition of the VFP fluorescence tag to the N terminus of ImuAʹ did not disrupt binding to ImuB (5). For SDS-PAGE analysis, we normalized the amount of ImuB loaded in the gel to infer the amount of ImuAʹ pulled down by each ImuB mutant. The amount of Strep-VFP–ImuAʹ pulled down in the IMAC column (HisTrap) *via* the HisTag on ImuB-L378A + V383A was substantially lower than the amount of ImuAʹ in complex with either of the single mutants (Fig. 3*C*).

The SEC profile for His-ImuB with Strep-ImuAʹ shows a main peak at 1.55 ml and a shoulder centered at 1.74 ml, which correspond to His-ImuB in complex with Strep-ImuAʹ and free His-ImuB (that is present after IMAC), respectively. The SEC profiles for all His-ImuB mutants displayed only a main peak that coincided with the shoulder for free His-ImuB (1.74 ml, Fig. 3*B*). This indicated that the single mutations also destabilized the interaction with Strep-ImuAʹ and, therefore, that complex formation was decreased relative to the wildtype. Following SEC, we analyzed the same fraction for all injected samples that coincided with the elution volume of His-ImuB–Strep-VFP–ImuA′ complex (∼1.6 ml) by SDS-PAGE (Fig. 3*C*). Strep-VFP–ImuAʹ was eluted together with His-ImuB, His-ImuB-L378A, and His-ImuB-V383A, confirming complex formation, albeit the amount of Strep-VFP–ImuAʹ detected for both single mutants was lower than that recovered by His-ImuB (Fig. 3, *B* and *C*). In contrast, Strep-VFP–ImuAʹ was absent in the same fraction of His-ImuB-L378A + V383A, consistent with a more impaired interaction between the double His-ImuB L378A + V383A mutant and ImuAʹ over time (Fig. 3*C*). These results align with lower amounts of Strep-VFP–ImuAʹ being pulled down by the His-ImuB single mutants during the IMAC pull-down than His-ImuB and even lower amounts pulled by His-ImuB L378A + V383A (Fig. 3*C*). Simultaneously, we measured the melting curves of the IMAC samples that yielded virtually the same melting temperature as His-ImuB–Strep-VFP–ImuAʹ sample (∼45 °C) (Fig. 3*D*). These results, together with the SEC data, demonstrate that the mutants are similarly folded to, and as thermostable as, wildtype His-ImuB; they also indicate that the weaker interaction between ImuA′ and ImuB observed in this double mutant is due to the loss of key interactions between ImuA′ and L378 and V383 of ImuB.

### RecA-NT homology motif within the ImuB C terminus is required for mutasome function

**Figure 4:**
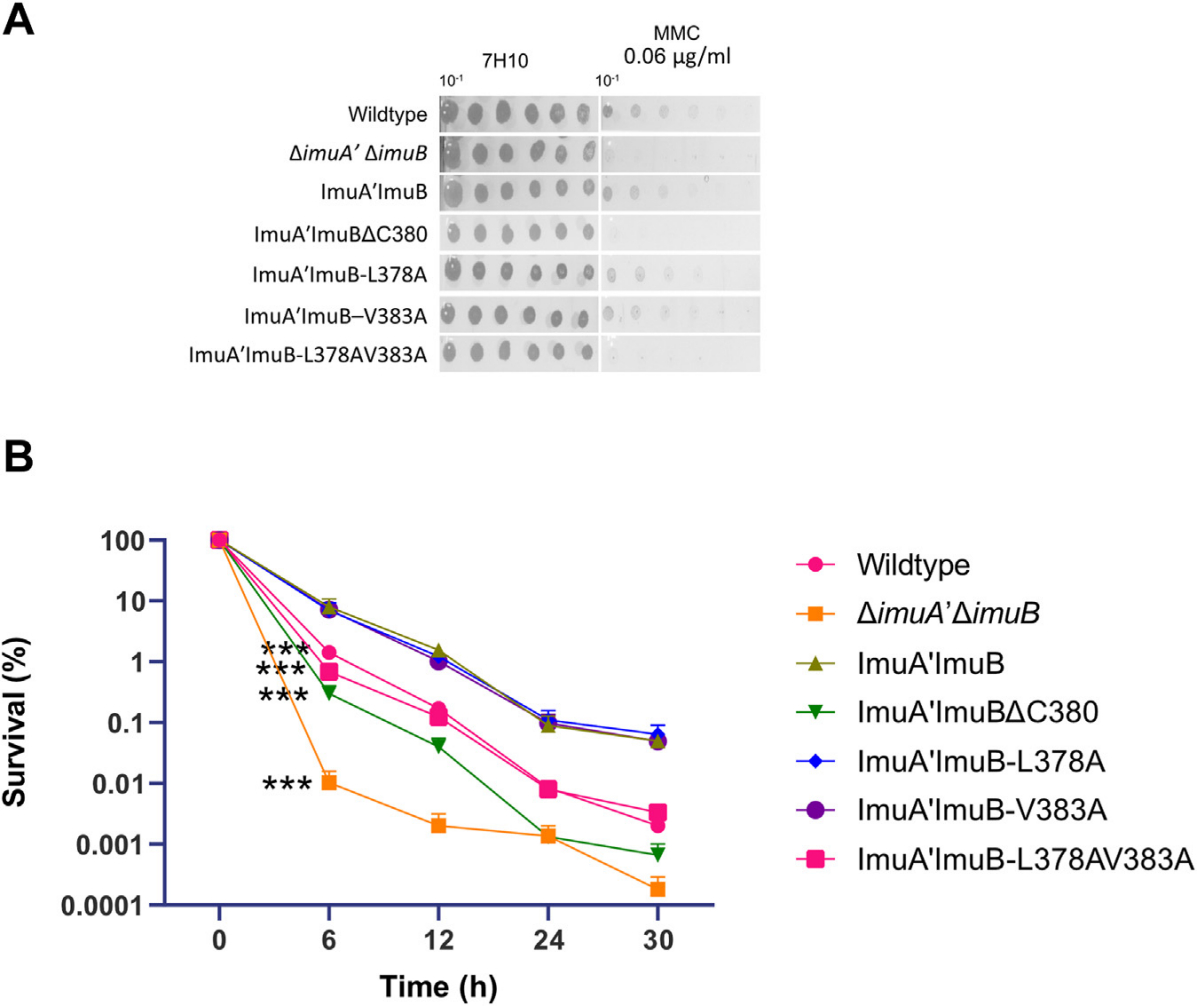
**Evaluation of functional complementation of mutant alleles by means of mitomycin C (MMC) sensitivity.***A*, MMC damage sensitivity assays in which a twofold dilution series of a 10-fold dilution of each *Mycobacterium smegmatis* culture (indicated by 10^−1^) was spotted on standard solid media (7H10) alone or supplemented with 0.06 μg/ml MMC. Images are representative of at least three biological repeats. Individual rows of spots were imaged from a single plate per treatment but represented as single rows for ease of presentation. *B*, MMC-kill curve showing survival (%) of the different strains exposed to 0.64 μg/ml MMC over a 30 h period. Survival was calculated based on the colony-forming units (CFUs)/ml at each time point divided by the CFU/ml at 0 h (prior to the start of treatment). The plot above represents the mean from three independent experimental repeats with the error bars representing the SD. ∗∗∗ indicate statistical significance at 6 h (*p* < 0.0001) compared with ImuA–ʹImuB using a two-way ANOVA with Tukey correction for multiple comparisons.

**Figure 5:**
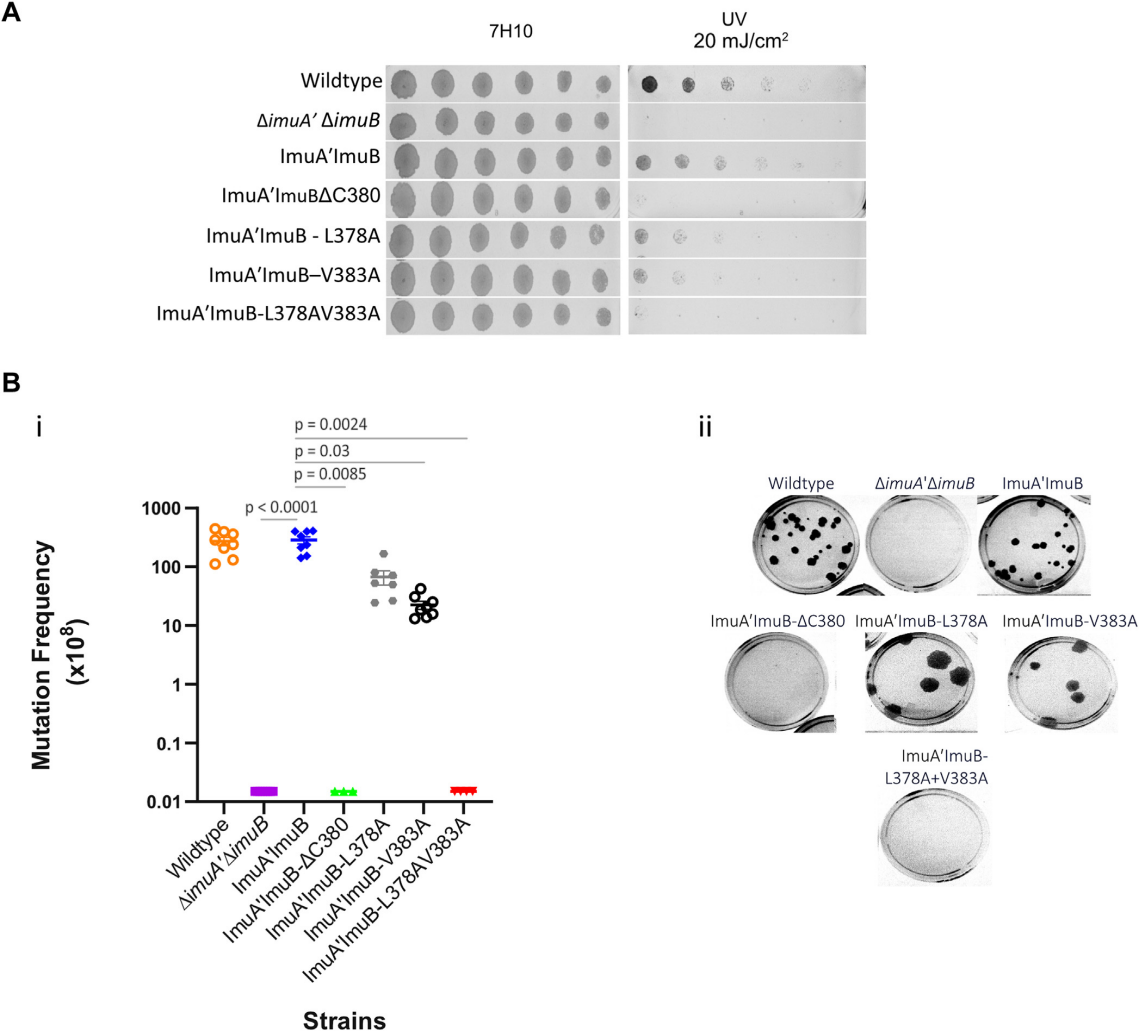
**Evaluation of functional complementation of mutant alleles by means of UV sensitivity and UV-induced mutagenesis.***A*, UV damage sensitivity assays were performed by spotting twofold dilutions of a 10-fold diluted (10^−1^) culture onto standard media. The spotted plates were then *left* unexposed (7H10) or exposed to 15 μJ/cm^2^ UV. Images are representative of three biological repeats. Individual rows of spots were imaged from a single plate per treatment but represented as single rows for ease of presentation. *B*, UV-induced mutagenesis assays were performed to calculate the mutation frequency of the different strains upon exposure to UV as measured by the appearance of rifampicin-resistant mutants. (i) Graph represents the mean mutation frequency of each strain from at least three experimental repeats with the error bars representing SD. A Kruskal–Wallis analysis with Dunn’s correction for multiple comparisons was done to determine statistical significance. (ii) A subset of rifampicin containing plates used to calculate the rifampicin-resistant colonies as a proxy for mutation frequency in (i) of each strain.

Finally, we asked which region within the C terminus of ImuB was required for mutasome function in live mycobacterial cells. For this purpose, we exploited a previously validated complementation system in which an integrative vector expressing the full-length *imuA*ʹ*imuB* operon from its native promoter is introduced into a Δi*muA*ʹΔ*imuB* double deletion mutant lacking both *imuAʹ* and *imuB* genes (2). Consistent with previous observations, the Δi*muAʹ*Δ*imuB* double knockout was hypersusceptible to mitomycin C (MMC) (Fig. 4) and UV (Fig. 5) and showed reduced UV-induced mutagenesis (Fig. 5*B*), which was restored by complementation with the wildtype *imuAʹimuB* locus. An exception was the MMC-kill curve, where complementation with the wildtype locus reduced sensitivity relative to the wildtype control (Fig. 4*B*). This could be because the complementation alleles (wildtype and mutants) are expressed from their native promoters on an integrative vector at the *attB* site in the bacterial genome and not at the native gene locus. Regardless, all mutant strains were generated in the wildtype ImuA'–ImuB complementation background, therefore ImuA'–ImuB was used as baseline for comparison. To confirm that the introduced alterations were not affecting protein expression and/or stability *in vivo*, selected mutations were introduced in a GFP-tagged ImuB in an *imuB* deletion mutant (5). Constant mean fluorescence across strains suggests that any lack of complementation was not because of reduced protein levels (Fig. S1).

To assess the contribution of the RecA-NT motif to ImuAʹ–ImuB interaction and mutasome function, we tested if ImuB-ΔC380, which lacks part of the RecA-NT motif and the downstream C-terminal region, retained mutasome function. The ImuB-ΔC380 deletion was incapable of complementing wildtype function in both MMC (Fig. 4*A*) and UV-spotting assays (Fig. 5*A*), phenocopying the Δ*imuAʹ*Δ*imuB* double knockout. Similarly, a statistically significant increase in sensitivity was observed in the MMC survival assays following 6 h of treatment (Fig. 4*B*). In addition, the UV damage–induced mutagenesis assays showed a significant reduction in UV-induced mutation frequency for ImuB-ΔC380 (Fig. 5*B*) compared with the ImuAʹ–ImuB complemented strain (*p* = 0.0085, Kruskal–Wallis with Dunn’s correction for multiple comparisons). In contrast to ImuB-ΔC380, the single mutations ImuB-L378A and ImuB-V383A had no apparent effect on the strains’ sensitivities to MMC compared with the parental ImuA'–ImuB strain (Fig. 4) and were associated with a subtle impact on the response to UV (Fig. 5), whereas the ImuB-L378AV383A double mutant phenocopied Δ*imuA*ʹΔ*imuB* and ImuB-ΔC380 in response to both MMC (Fig. 4) and UV (Fig. 5). A similar UV sensitivity pattern was observed for the GFP-tagged variants (Fig. S1), supporting these observations. The inability of the ImuB-ΔC380 deletion mutant or the ImuB-L378AV383A double mutant to complement wildtype function in any of the four microbiological assays therefore indicates that the ImuA′–ImuB complex formation is also required for mutasome activity in cells.

## Discussion

A full complement of intact functional mutasome proteins is required for induced mutagenesis and DNA damage survival in mycobacteria (2). Although the precise role of ImuB in mutasome function remains elusive, multiple lines of evidence support its importance in enabling key interactions with (and perhaps between) the other mutasome components, ImuAʹ and DnaE2 (2, 5). However, despite early evidence suggesting the importance of the ImuB C terminus in mediating these interactions, no detail has been provided about the specific regions, motifs, or residues involved. Previous computational analyses identified two conserved regions in the C terminus of ImuB:RecA-NT, located immediately downstream of the β clamp-binding site (7), and ImuB-C, which is just downstream of RecA-NT (7, 14; Fig. 1*D*). Based on its homology to the N-terminal region of *E. coli* RecA—which is required for its oligomerization—we hypothesized that the RecA-NT region in the C terminus of ImuB might facilitate interaction with ImuAʹ given its distant homology to RecA, its essential role in mutasome function, and the interaction between ImuAʹ and ImuB inferred from Y2H studies (2). By designing a panel of truncations and site-directed mutants of ImuB, we have presented experimental evidence confirming the essential role of the RecA-NT motif in the ImuAʹ–ImuB interaction.

The RecA-NT motif was identified based on homology to the region in *E. coli* RecA, which facilitates interaction with an upstream RecA molecule in the RecA filament (7). The presence of this motif in the C-terminal region of Y-family polymerases suggests a widespread interplay between RecA and these polymerases. This is not surprising since it has long been known that the *E. coli* PolV mutasome (UmuDʹ_2_C) requires UmuC (a Y-family polymerase) to bind an RecA monomer in the RecA filament as the first step toward activation. Following binding of RecA and ATP, the PolV multiprotein complex (UmuDʹ_2_C-RecA-ATP) is catalytically active (15, 16). In addition to *E. coli*, functionality of the *Bacillus subtilis* Y-family polymerase, PolY2 (YqjW), also showed dependence on RecA (17).

An AlphaFold structural model of the ImuAʹB complex shows similarity to the RecA–RecA binding (Fig. 1). At the same time, there are some differences. The ImuAʹ–ImuB interaction interface appears to be more extensive than the RecA–RecA interface (Fig. 1, Table S2). The C-terminal region of ImuB beyond RecA-NT (ImuB-C) is also predicted to be involved in binding ImuAʹ, contributing similar surface area to the interaction as that of RecA-NT (Table S3). Nonetheless, the ImuB RecA-NT motif appears to be the key element for binding ImuAʹ, because disrupting the hydrophobic residues in RecA-NT almost completely abolished ImuAʹ–ImuB interaction (Fig. 3) and mutasome function (Figs. 4, 5). In further support of this conclusion, an ImuB-ΔC433 truncation, which preserves RecA-NT but eliminates ImuB-C, retained ImuAʹ binding ability in biochemical assays (Fig. 2), whereas deletion of both ImuB-C and part of RecA-NT fully compromised the interaction (Fig. 2). This result is in agreement with a recent study in *M. xanthus* that identified the region in ImuB responsible for interaction with ImuAʹ to be residues 339 to 367, which includes the RecA-NT motif (P346–L363) (8).

Given the apparently central role of RecA-NT in the interaction between ImuB and ImuAʹ (similar to how UmuC binds RecA), it is tempting to speculate that, by binding ImuB, ImuAʹ might play a structural role analogous to RecA in *E. coli* PolV; however, this remains to be demonstrated. In addition to ImuAʹ, it seems reasonable to expect that RecA-NT might mediate interactions with other RecA homologs; for example, RadA and SulA, depending on the presence of a functional binding site for RecA-NT. However, the biological significance of such interactions remains unclear, and the ability of RecA-NT to interact with other RecA homologs requires experimental validation. In addition, at least in some cases, RecA-NT might mediate the interaction between RecA and ImuAʹ as was recently demonstrated in *M. xanthus*, which seemingly suppresses the homologous recombination activity of RecA, stimulating mutasome function (9). Considering other interacting partners of ImuB in the mutasome, it is unlikely that RecA-NT plays a role in the interaction between ImuB and DnaE2 given that RecA-NT is already occupied by interacting with ImuAʹ, and even if available, DnaE2, unlike ImuAʹ, lacks the region homologous to the RecA core, to facilitate the interaction with RecA-NT.

The potential importance of the mycobacterial mutasome—minimally comprising ImuAʹ, ImuB, and DnaE2—in *M. tuberculosis* evolution and acquisition of drug resistance has been recognized previously (1, 2, 18, 19). We are optimistic that the results presented will allow for a better understanding of the assembly and operation of this mutagenic machinery and will inform ongoing efforts toward developing novel “antievolution” drugs (20, 21, 22, 23) for *M. tuberculosis* and other organisms encoding homologous systems.

## Experimental procedures

### Computational modeling and analysis

A structural model of the complex formed by the full-length ImuAʹ (National Center for Biotechnology Information ID: ABK74665.1) and ImuB (National Center for Biotechnology Information ID: ABK76097.1) was generated using a local installation of AlphaFold-Multimer v2 (11, 12) with default parameters. The ImuA′ N terminus (residues 1–64) in the model appeared unstructured and was removed from subsequent analyses. Structural analyses and visualizations were performed using UCSF Chimera (24). Residue–residue contacts at the interaction interface were analyzed using VoroContacts (25).

### Oligonucleotides and chemicals

All chemicals were obtained from Sigma–Aldrich unless otherwise stated, and oligonucleotides used for cloning and sequencing were purchased from Integrated DNA Technologies. A full list of plasmids used in this study is given in Table S1.

### Bacterial strains and growth conditions

*E. coli* strain DH5α was used for cloning procedures, and BL21(DE3) was used for recombinant protein expression. All *E. coli* strains were grown in LB culture medium at 37 °C shaking, with the addition of kanamycin (50 μg/ml) and/or streptomycin (50 μg/ml) where appropriate. All *M. smegmatis* strains were cultured in standard Difco Middlebrook 7H9 (BD Biosciences) supplemented with 10% BBL Middlebrook OADC Enrichment (BD Biosciences), 0.2% glycerol (v/v), and 0.05% Tween-80 at 37 °C, shaking. For propagation on solid agar plates, Difco Middlebrook 7H10 (BD Biosciences) was supplemented with 0.5% glycerol (v/v). Kanamycin (20 μg/ml) was added to liquid and solid growth media where appropriate. Solid plates were incubated at 37 °C for 3 to 5 days, unless stated otherwise. *M. smegmatis* wildtype (mc^2^155) (26) was maintained by culturing in standard 7H9, whereas Δ*imuAʹ*Δ*imuB* (2) and Δ*imuB* expressing the complemented and mutant alleles were maintained in 7H9 supplemented with kanamycin.

### Mutant allele generation

C-terminal deletions were generated by PCR by deleting the region downstream of the target site. Site-directed mutations were introduced using the Q5 site-directed mutagenesis kit (New England BioLabs) as per the manufacturer’s protocol. Primers were designed using NEBaseChanger (https://nebasechanger.neb.com/). Mutant constructs were confirmed with Sanger sequencing and introduced into Δ*imuAʹ*Δ*imuB* as per the standard electroporation protocol (27). Kanamycin-resistant colonies were selected and confirmed by Sanger sequencing. Site-directed mutants of GFP-ImuB were constructed as described previously in an N-terminal GFP-tagged ImuB (5). Mutant constructs were confirmed with Sanger sequencing and introduced into a Δ*imuB* background. Kanamycin-resistant colonies were selected and confirmed using Sanger sequencing.

### GFP-ImuB site-directed mutant expression comparison

The suite of strains expressing GFP-ImuB and the site-directed mutants of GFP-ImuB (L378A, V383A, L378AV383A, and ΔC380) were grown to an absorbance of ∼0.2 at 600 nm. Cultures were treated and imaged as previously described (5). Briefly, each culture was treated with 0.16 μg/ml MMC for 4 h to induce expression of ImuB, following which the cells were imaged using a Zeiss Axio Observer Z1. Images of the different strains were captured with the same instrument and exposure settings. Green fluorescence was detected using the Zeiss Filter Set 38 HE. Images were processed, and fluorescent signal was quantified using the MicrobeJ (28) plugin of ImageJ (29). A Kruskal–Wallis test was done to compare the mean fluorescence per cell between strains.

### Damage-sensitivity spotting assays

For damage-sensitivity spotting assays, the cultures were grown to an absorbance of ∼0.2 at 600 nm, following which a twofold dilution series was prepared from a 10-fold dilution of the culture. About 5 μl of the twofold dilution series was spotted on standard 7H10 medium and 7H10 medium supplemented with 0.06 μg/ml MMC. For UV sensitivity, the twofold dilution series of a 10-fold diluted culture was spotted onto 7H10 plates and treated with UV (20 mJ/cm^2^). Plates were incubated for 3 days and imaged.

### MMC-kill curve

Cultures were grown to an absorbance of ∼0.2 at 600 nm, following which the cultures were exposed to 0.65 μg/ml MMC for 30 h. A small aliquot was removed from the treated samples at 0, 6, 12, 24, and 30 h after the start of treatment and used to prepare a 10-fold serial dilution for colony-forming unit (CFU)/ml calculation. Percentage strain survival was calculated by dividing the CFU/ml at the respective time points (6, 12, 24, and 30 h) by CFU/ml calculated at baseline (0 h). A two-way ANOVA with Tukey correction for multiple comparison was done to determine whether the strains’ survival was significantly different at each time point.

### UV-induced mutagenesis

UV-induced mutagenesis assays were performed as previously described (1, 2, 5). Briefly, a log-fold serial dilution of midlog phase cultures was prepared and plated on standard media for CFU/ml determination prior to UV treatment. The remaining culture was concentrated *via* centrifugation and exposed to 250 mJ/cm^2^ UV. Fresh standard media were added following UV exposure to make up the original volume, and cultures were left to recover at 37 °C for 3 h, shaking. Following recovery, 1 ml of the culture was plated onto standard media supplemented with rifampicin (200 μg/ml). In addition, a log-fold dilution of the post-UV cultures was also plated on standard media to determine CFU/ml post-UV treatment. Standard media plates for CFU/ml calculation were incubated for 3 days at 37 °C. Rifampicin-resistant (Rif^R^) colonies were counted after 5 days of incubation at 37 °C. The mutation frequency was calculated as follows: $\frac{RifRcolonycount}{\frac{CFU}{ml}prior\;UV\;treatment}$

A Kruskal–Wallis analysis with Dunn’s correction for multiple comparison was done to determine whether mutation frequency differences between strains were statistically significant.

### Protein expression and purification

*M. smegmatis imuAʹ* and *imuB* were cloned into ligation-independent cloning vectors Lic1 (kanamycin resistance, N-terminal His tag) and Lic6 (streptomycin resistance, N-terminal Strep tag), respectively (30). Coexpression of ImuB, both wildtype (His-ImuB) and mutant forms (His-ImuB-L378A, His-ImuB-V383A, and His-ImuB-L378A + V383A), with Strep-ImuAʹ (and Strep-VFP–ImuAʹ) was performed using *E. coli* BL21(DE3) cells. Briefly, cells were grown in 2xYT medium supplemented with 1 mM magnesium sulfate, 50 μg/ml kanamycin, and 50 μg/ml streptomycin. Protein expression was induced with 1 mM IPTG, and cells were grown for 3 h at 30 °C. Clarified protein extracts were either loaded onto a HisTrap HP column (Cytiva Life Sciences) for double pull-down experiments or directly incubated with nickel beads for analysis by SEC; His-tagged ImuB was either eluted with an imidazole gradient or with 500 mM imidazole, respectively. In pull-down experiments, fractions containing protein were then pooled and loaded onto StrepTrap HP columns (Cytiva Life Sciences) and eluted with 5 mM desthiobiotin. All IMAC and affinity purification steps were carried out in 50 mM Tris (pH 8.5), 0.3 to 0.5 M NaCl, and samples were analyzed by SDS-PAGE electrophoresis using 12% Bolt Bis–Tris precast gels (Invitrogen).

### Size-exclusion chromatography

Samples were injected either onto a Superdex 200 PC 3.2/300 (Cytiva Life Sciences) or a Superdex 200 10/300 GL columns equilibrated in 50 mM Tris (pH 8.5), 300 mM NaCl, and 1 mM DTT; thereafter, 50 μl or 500 μl fractions were collected, respectively.

### Thermal protein melt

Protein melting curves were measured in UV capillaries using the Tycho NT6 (NanoTemper Technologies) where protein unfolding is followed by detecting the fluorescence of intrinsic tryptophan and tyrosine residues at both emission wavelengths of 350 and 330 nm during a thermal ramp from 35 °C to 95 °C at a defined rate of 30 °C/min. The data are automatically analyzed by the equipment software to calculate inflection temperatures (T_i_) at which the protein unfolds.

### SDS-PAGE analysis

Protein samples were mixed with Laemmli sample buffer before loading onto 12% Bolt Bis–Tris precast gels (Invitrogen) and subjected to electrophoresis in Mes–SDS running buffer. For VFP-ImuA′ detection, gels were scanned using a Typhoon FLA 9500 (GE Healthcare Life Sciences) with a 532 nm excitation laser with a resolution of 100 microns before staining with Coomassie. All Coomassie-stained SDS-PAGE gels were visualized using a GelDoc XR+ imager and the ImageLab software from Bio-Rad.

### Statistical analyses

Data were tested for normality using Shapiro–Wilk Test. The survival of strains in the MMC-kill curve was compared over time using a two-way ANOVA with Tukey correction for multiple comparisons. *p* Values in Figure 4 are recorded based on comparisons with the ImuA'–ImuB strain. Comparison of mutation frequencies was analyzed using a Kruskal–Wallis test with Dunn’s correction for multiple comparisons.

## Data availability

The data underlying this article are available in the article and its online supporting information. Where additional information or raw data are needed, the data will be shared on reasonable request to the corresponding author.

## Supporting information

This article contains supporting information (2, 5, 26, 30, 32).

## Conflict of interest

The authors declare that they have no conflicts of interest with the contents of this article.
