# Supplementary material for: The RecA-NT homology motif in ImuB mediates the interaction with ImuA′, which is essential for DNA damage–induced mutagenesis
Source: J Biol Chem. 2024 Dec 18;301(2):108108. doi: 10.1016/j.jbc.2024.108108 (PMC11791113; doi:10.1016/j.jbc.2024.108108)
Supplement: Supporting information [file mmc1.docx]

**Supplementary Information**

**The RecA-NT homology motif in ImuB mediates the interaction with ImuA' which is essential for DNA damage-induced mutagenesis**

Joana A. Santos^1^, Kęstutis Timinskas^2^, Atondaho A. Ramudzuli^3,4^, Meindert H. Lamers^1^, Česlovas Venclovas^2^, Digby F. Warner^3,4,5^, Sophia J. Gessner^3,4,*^

^1^Department of Cell and Chemical Biology, Leiden University Medical Center, The Netherlands;

^2^Institute of Biotechnology, Life Sciences Center, Vilnius University, Saulėtekio 7, Vilnius LT-10257, Lithuania;

^3^SAMRC/NHLS/UCT Molecular Mycobacteriology Research Unit, DSI/NRF Centre of Excellence for Biomedical TB Research, Department of Pathology, University of Cape Town, South Africa;

^4^Institute of Infectious Disease and Molecular Medicine, University of Cape Town, South Africa;

^5^Wellcome Centre for Infectious Diseases Research in Africa, University of Cape Town, South Africa.


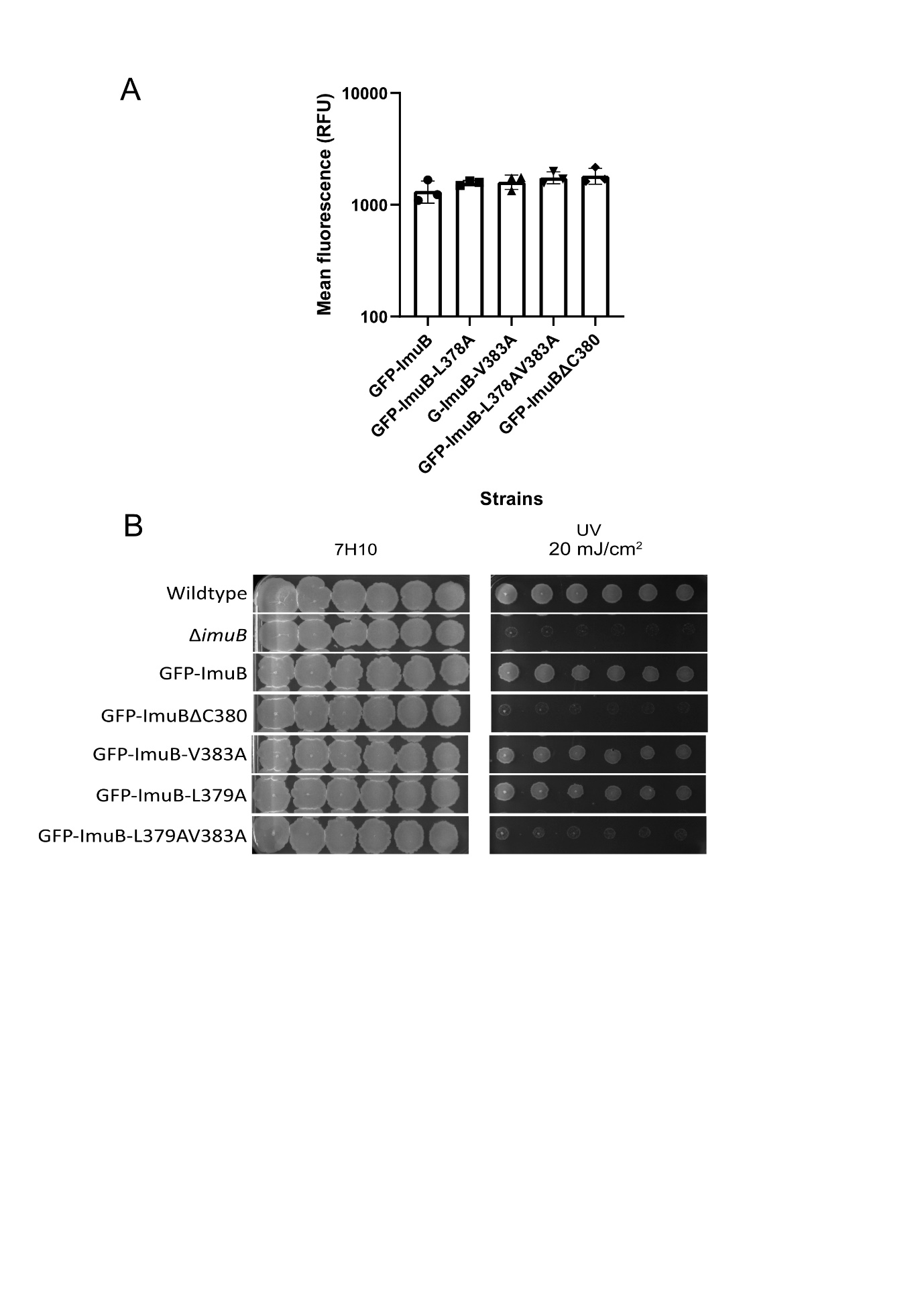


**Fig. S1. GFP-tagged ImuB variants.** A. The mean fluorescence of GFP-ImuB expressing strains with site directed mutations L378A, V383A, L378AV383A and ΔC380 were measured following treatment with mitomycin C. On average 80 bacterial cells were analyzed at random per strain per experiment. Three independent experiments were conducted with the error-bars representing standard deviation between experiments. The differences between the strains’ mean GFP expression are not statistically significant (Kruskal-Wallis test, p=0,3220). B. UV damage sensitivity spotting assays where two-fold dilutions of a neat culture spotted on standard media (7H10) and left either untreated (left panel) or treated with UV (20 mJ/cm^2^). Images are representative of two repeats. Individual rows of spots were imaged from a single plate per treatment but are presented as single rows for ease of presentation.

**Table S1. Vectors used in this study. Strep^R^= streptomycin resistance, Kan^R^= kanamycin resistance**

| **Plasmid name** | **Used for** | **Description** | **Reference** |
| --- | --- | --- | --- |
| ***E. coli* vectors** | | | |
| pETNKI-his-3C-LIC-kan | For production of *M. smegmatis* ImuB *wildtype* and mutant forms | pET vector backbone (Kan^R^) with NKI LIC cassette with N-terminal His6 tag and 3C-preScission cleavage site to remove His6 tag. | (Luna-Vargas et al. 2011) |
| pCDFNKI-strepII-3C-LIC-strep | For production of *M. smegmatis* ImuA’ *wildtype* and VFP tagged | pCDF vector backbone (Strep^R^) withNKI LIC cassette containing N-terminal StrepII tag and 3C-preScission cleavage site to remove StrepII tag. |  |
| ***M. smegmatis* vectors** | | | |
| pMCAINT::*imuAʹimuB* | Introducing targeted mutations/deletions to complement the Δ*imuAʹ*Δ*imuB* deletion strain. | pAINT vector backbone (Kan^R^) expressing *imuAʹ* and *imuB* from the native promoter. | (Warner et al. 2010) |
| pMCAINT::GFP-ImuB | Introducing targeted mutations/deletions GFP tagged ImuB. | pAINT vector backbone (Kan^R^) expressing and N-terminal GFP tagged *imuB* from the native promoter. | (Gessner et al. 2023) |

**Table S2. Strains used in this study. Kan^R^= kanamycin resistance**

| **Strain name** | **Description** |  |
| --- | --- | --- |
| *E.coli* strains | | |
| BL21(DE3) | Wildtype | (Studier and Moffatt 1986) |
| *M. smegmatis* strains | | |
| mc^2^155 | Wildtype | (Snapper et al. 1990) |
| Δ*imuAʹ*Δ*imuB* | Double deletion mutant of *imuAʹ* and *imuB* | (Warner et al. 2010) |
| Δ*imuAʹ*Δ*imuB*::ImuAʹImuB | Double deletion mutant of *imuAʹ* and *imuB* complemented with the wildtype *imuAʹ-imuB* operon at the *attB* site. Kan^R^. | (Warner et al. 2010) |
| Δ*imuAʹ*Δ*imuB*::ImuAʹImuB-L378A | Double deletion mutant of *imuAʹ* and *imuB* complemented with the *imuAʹ-imuB* operon with ImuB-L378A at the *attB* site. Kan^R^. | This study |
| Δ*imuAʹ*Δ*imuB*::ImuAʹImuB-V383A | Double deletion mutant of *imuAʹ* and *imuB* complemented with the *imuAʹ-imuB* operon with ImuB-V383A at the *attB* site. Kan^R^. | This study |
| Δ*imuAʹ*Δ*imuB*::ImuAʹImuB-L378AV383A | Double deletion mutant of *imuAʹ* and *imuB* complemented with the *imuAʹ-imuB* operon with ImuB-L378AV383A at the *attB* site. Kan^R^. | This study |
| Δ*imuAʹ*Δ*imuB*::ImuAʹImuB-ΔC380 | Double deletion mutant of *imuAʹ* and *imuB* complemented with the *imuAʹ-imuB* operon with a C-terminal deletion of ImuB (ΔC380) the *attB* site. Kan^R^. | This study |
| GFP-ImuB | Δ*imuB* complemented with a GFP-ImuB. Kan^R^. | (Gessner et al. 2023) |
| GFP-ImuB-L378A | GFP-ImuB with L378A mutations. Kan^R^. | This study |
| GFP-ImuB-V383A | GFP-ImuB with V383A mutations. Kan^R^. | This study |
| GFP-ImuB-L378AV383A | GFP-ImuB with L378AV383A mutations. Kan^R^. | This study |
| GFP-ImuB-ΔC380 | GFP-ImuB with ImuB C-terminal truncation. Kan^R^. | This study |

**Table S3. Interface surface areas between ImuB constructs and full length ImuAʹ**. Calculated with VoroContacts from the ImuA’-ImuB AlphaFold model.

| **ImuB construct** | **Interface area with ImuA’, Å^2^** |
| --- | --- |
| ImuB, full | 1804 |
| ImuB, 360-388 (RecA-NT motif) | 694 |
| ImuB, 422-525 (ImuB-C) | 960 |
| ImuB, 1-359 (C-terminal region removed) | 0 |

**References**

Gessner, Sophia et al. 2023. “Investigating the Composition and Recruitment of the Mycobacterial ImuA′– ImuB–DnaE2 Mutasome.” *eLife* 12.

Luna-Vargas, Mark P.A. et al. 2011. “Enabling High-Throughput Ligation-Independent Cloning and Protein Expression for the Family of Ubiquitin Specific Proteases.” *Journal of Structural Biology* 175(2): 113–19.

Snapper, S. B. et al. 1990. “Isolation and Characterization of Efficient Plasmid Transformation Mutants of Mycobacterium Smegmatis.” *Molecular Microbiology* 4(11): 1911–19. http://doi.wiley.com/10.1111/j.1365-2958.1990.tb02040.x (November 10, 2016).

Studier, F. William, and Barbara A. Moffatt. 1986. “Use of Bacteriophage T7 RNA Polymerase to Direct Selective High-Level Expression of Cloned Genes.” *Journal of Molecular Biology* 189(1): 113–30.

Warner, Digby F. et al. 2010. “Essential Roles for ImuA′- and ImuB-Encoded Accessory Factors in DnaE2-Dependent Mutagenesis in Mycobacterium Tuberculosis.” *Proceedings of the National Academy of Sciences of the United States of America* 107(29): 13093–98.
